# Modulation of corticosterone and changes of signal molecules in the HPA axis after cold water swimming stress

**DOI:** 10.1080/19768354.2021.1890211

**Published:** 2021-02-23

**Authors:** Jing Hui Feng, Su Min Sim, Jung Seok Park, Jae Seung Hong, HongWon Suh

**Affiliations:** a Department of Pharmacology and Institute of Natural Medicine, College of Medicine, Hallym University, Chun-Cheon, South Korea; b Department of Physical Education, Hallym University, Chun-Cheon, South Korea

**Keywords:** Corticosterone, HPA axis, signal molecules

## Abstract

In the present study, we examined the effect of cold-water swimming stress (CWSS) on plasma corticosterone levels. Mice were exposed to stress in 4°C for 3 mins. Plasma corticosterone (CORT) level was measured at 0, 15, and 30 min after stress stimulation. The plasma CORT level was gradually increased up to 30 min. Then we further examined the changes of several signaling molecules expression levels, such as p-ERK, p-JNK, p-P38, p-AMPKα1, p-AMPKα2, and p-mTOR, in the HPA axis. We observed that those signaling molecules were altered after stress in the HPA axis. p-ERK, p-JNK, p-P38, and p-mTOR proteins expression were reduced by CWSS in the HPA axis. However, the phosphorylation of AMPKα1 and AMPKα2 were activated after CWSS in the HPA axis. Our results suggest that the upregulation of plasma CORT level induced by CWSS may be modulated by the those signaling molecules.

## GRAPHICAL ABSTRACT

**Figure:**
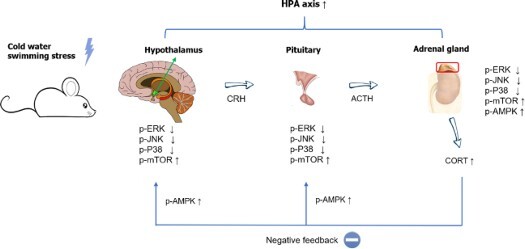

## Introduction

Stress is recognized as an adaptive response of an organism to stimulus in the environment, aiming to re-establish homeostasis (Micale and Drago 2018; Park et al. 2020). Stress elicits the activation of the hypothalamic-pituitary-adrenocortical (HPA) axis, which results in the release of corticosterone (CORT) in rodents from the adrenal cortex into the bloodstream as the index of neuroendocrine response, and the activation of the sympathetic-adrenergic system, which culminates in the release of adrenaline and noradrenaline from the adrenal medulla and nerve terminals respectively, into the blood circulation, as the index of autonomic response (Lowrance et al. 2016; Kinlein et al. 2019; Hueston and Deak 2020). Once a CORT response has been mounted following stressor termination, the HPA axis is reset by negative feedback, which occurs mainly at the paraventricular nucleus (PVN) and pituitary (De Kloet et al. 2005; Armario 2006). Glucocorticoids (GCs) elicited primarily their effects on transcription, a process responsible for the delayed but not for the fast feedback HPA axis regulation, which occurs within minutes after stressor exposure (Dallman 2005). Although the molecular mechanisms underlying the mechanisms of feedback regulation of the HPA axis are not fully understood it seems that CORT could be involved in this feedback process.

Cold water swimming stress (CWSS), one type of stress, has been studied for decades. Previous researchers focused on the analgesic effect induced by CWSS in the mice (Terman et al. 1986; Choi et al. 2003). Recent years, a series of studies evaluated the physiological response after CWSS, either in human or in animals. For example, pre-treatment of CWSS showed a protective effect from brain injury in rats (Zhou et al. 2017). Regular CWS lead to adaptive changes in the blood elements, including serum erythropoietin (EPO), erythrocyte superoxide dismutase (SOD), and glutathione (GSH) (Checinska-Maciejewska et al. 2019; Lubkowska et al. 2019). We also reported that the blood glucose level was increased after CWSS (Suh et al. 2016). However, the relation between CWSS and CORT along with the mechanism has not been determined.

There are some pathways essential for stress response, such as mitogen-activated protein kinase (MAPK) and AMP-activated protein kinase (AMPK) pathways (Kyriakis and Avruch 2012; Kazyken et al. 2019). For example, acute swim stress leads to the activation of ERK and JNK MAPK pathways in rat brain regions (Shen et al. 2004). Moreover, some previous studies reported that MAPK and AMPK pathways were involved in the regulation of cold stress (Zhang et al. 2014; Galic et al. 2018; Xu et al. 2019). Additionally, CORT is reported as a factor in the regulation of MAPK and AMPK pathways in PC12 cells (Sun et al. 2008; Gong et al. 2019; Ma et al. 2020). Although the regulation of some signaling molecules in some stress models has been characterized, the relationship between the CORT level and the signaling proteins expression level in the CWSS model has not been well studied yet. Thus, in the present study, we aimed to examine the changes in plasma CORT level and the signaling proteins expression level in the HPA axis after CWSS.

## Materials and methods

These experiments were approved by the Hallym University Animal Care and Use Committee (Registration Number: Hallym R1 2017–57). All procedures were conducted in accordance with the ‘Guide for Care and Use of Laboratory Animals’ published by the National Institutes of Health and the ethical guidelines of the International Association for the Study of Stress.

### Experimental animals

Male ICR mice (MJ Co., Seoul, Korea) weighing 20∼25 g were used for all the experiments. Animals were housed 5 per cage in a room maintained at 22 ± 0.5°C with an alternating 12 h light–dark cycle. Food and water were available ad libitum. The animals were allowed to adapt to the laboratory for at least 2 h before testing and were only used once. Experiments were performed during the light phase of the cycle (10:00∼17:00).

### Cold-water swimming stress model

The mice were forced to swim in cold (4°C) water for 3 min. The mice were allowed to swim in a container 15 cm in diameter and 20 cm tall with water filled to a depth of 11 cm. After the swimming, the mice were gently dried by patting the body with a paper towel.

### Corticosterone assay

The plasma CORT level was determined by the fluorometric determination method (Glick et al. 1964). Four hundred microliters of blood were collected by puncturing the retro-orbital venous plexus. Plasma was separated by centrifugation (8000 rpm, 15 min, 4°C) and stored at −80°C until assayed.

### Protein extraction and western blot

Western blot analysis was carried out as previously described (Hong et al. 2020). Briefly, after treating cold water swimming stress (4°C, 3 min) for 0, 15, and 30 min the hypothalamus, pituitary, and adrenal gland were collected and washed three times with cold TBS, immediately frozen and stored in the ultra-lower temperature refrigerator (−80°C) until assay. The dissected tissues were lysed with sodium dodecyl sulfate lysis buffer. The sample was then centrifuged at 13,000 rpm for 15 min at 4°C, and the supernatant was retained. Protein concentrations were evaluated with the Bradford method (Bio-Rad Laboratories, Hercules, CA, USA) using bovine serum albumin as the standard. The samples were boiled after adding bromophenol blue (0.1% w/v). Equal amounts of protein (10–20 μg) were resolved by 6–10% SDS-polyacrylamide gel electrophoresis system and transferred to a polyvinylidene difluoride membrane (Millipore, Bedford, MA, USA). After blocking (2 hr at room temperature) with 5% skim milk in TBS containing 0.4% Tween-20, the membranes were immunoblotted with antibodies p-ERK (Cell Signaling Technology, 1:1000), p-P38 (Cell Signaling Technology, 1:1000), p-JNK (Cell Signaling Technology, 1:1000), p-AMPKα1 (Abcam, 1:1000), p-AMPKα2 (Abcam, 1:1000), p-mTOR (Cell Signaling Technology, 1:1000), and *β*-actin (Cell Signaling Technology, 1:1000) in a blocking buffer for overnight at 4°C. Then the membranes were washed 4 times with TBST for 20 min and incubated with the anti-rabbit IgG-horseradish peroxidase conjugated secondary antibody (Enzo Life Sciences, 1:4000) in blocking buffer at room temperature for 1 h. After washing the membranes with TBST for 20 min (4 times), the antibody–antigen complexes were detected using the ECL system and exposed to Luminescent Image Analyzer (LAS-4000, Fuji Film Co., Japan) for the detection of light emission. Those antibodies’ band densities were evaluated from the respective band densitometry. The Multi-Gauge Version 3.1 (Fuji Film Co., Japan) was used to analyze the intensity of expression. These values were expressed as the percentage of the control tested protein/*β*-actin for each sample.

### Statistical analysis

Statistical analysis was carried out by ANOVA (Bonferroni test) for multiple comparisons and student t-test by using GraphPad Prism Version 8.0.2 (GraphPad Software, San Diego, CA, USA). *p*-values less than 0.05 were considered to indicate statistical significance. All values were expressed as the mean ± SEM.

## Results

### Effect of CWSS on plasma CORT level.

**Figure 1.**
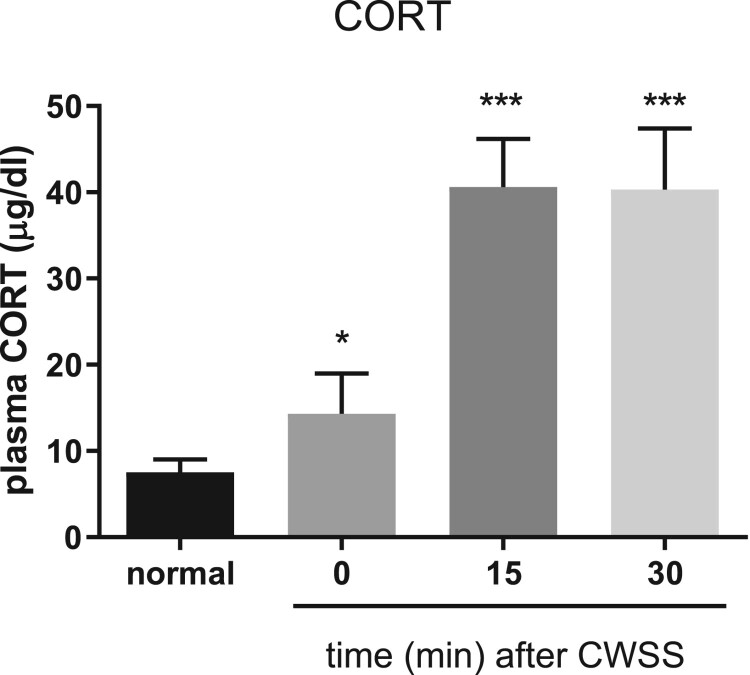
Effect of acute CWSS on plasma CORT. The plasma CORT levels were examined for 0, 15, and 30 min after cold water swimming stress. Values are mean ± SEM. The mice number of animals used in each group was 8. *p < 0.05, **p < 0.01, ***p < 0.001.

Mice were forced into CWSS for 3 min. Then the blood was collected. The plasma CORT level was gradually increased a fter 3 min acute CWSS, as shown in Figure 1. After CWSS, the plasma CORT level was immediately up-regulated. Moreover, CWSS continuously enhanced the secretion of CORT from 15 min to 30 min.

### Effect of CWSS on ERK phosphorylation in the HPA axis

**Figure 2.**
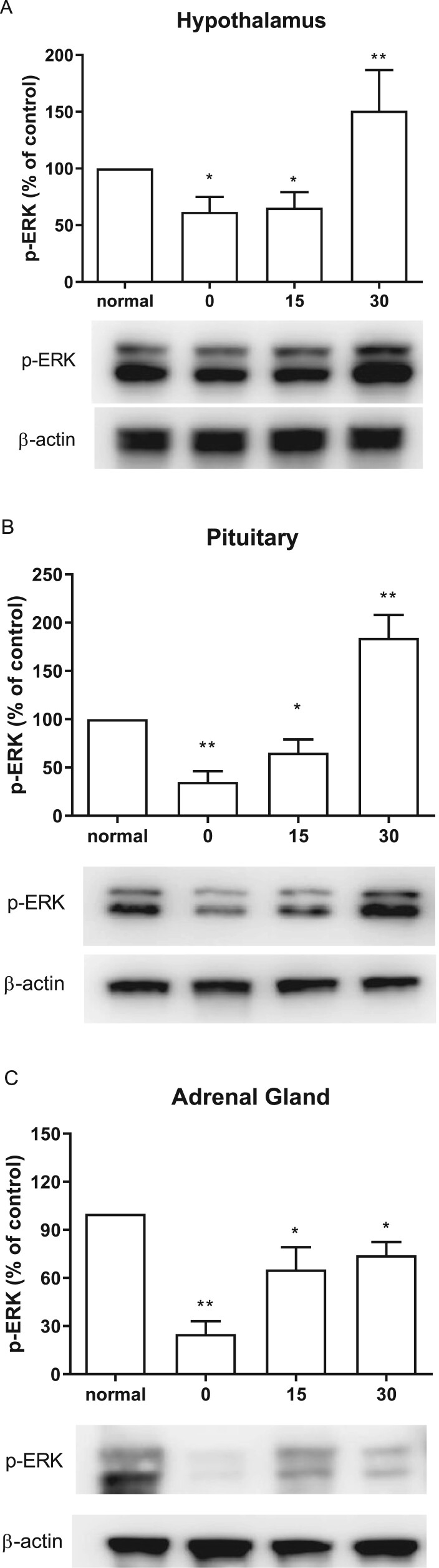
Effect of acute CWSS on ERK protein expression in the HPA axis. The protein p-ERK expression in the hypothalamus (A), in the pituitary (B), and the adrenal gland (C) were analyzed by Western blot. *β*-Actin (1:1000 dilution) was used as an internal loading control. Signals were quantified with the use of laser scanning densitometry and expressed as a percentage of the control. Values are mean ± SEM. The number of animals in each group was 6. *p < 0.05, **p < 0.01, ***p < 0.001.

The expression of ERK phosphorylation was evaluated by western blotting. The hypothalamus, pituitary, and adrenal gland were dissected at 0, 15, 30 mins after cold water swimming stress. CWSS caused down-regulation of p-ERK in the HPA axis (Figure 2). Both in the hypothalamus and pituitary, CWSS suppressed the expression level of p-ERK, which remained lower than that of the normal level at 15 min, whereas CWSS significantly increased p-ERK level at 30 min (Figure 2(A and B)). However, the ERK phosphorylation level was decreased up to 30 min after CWSS in the adrenal gland (Figure 2(C)).

### Effect of CWSS on the expression of p-JNK in the HPA axis

**Figure 3.**
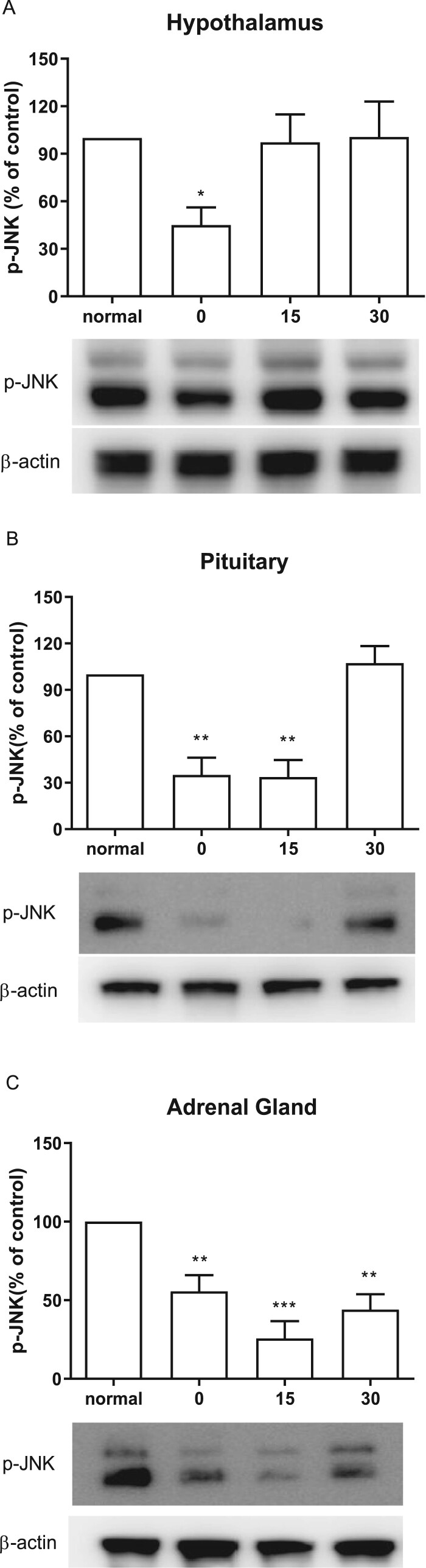
Effect of acute CWSS on JNK protein expression in the HPA axis. The protein p-JNK expression in the hypothalamus (A), in the pituitary (B), and the adrenal gland (C) were analyzed by Western blot. *β*-Actin (1:1000 dilution) was used as an internal loading control. Signals were quantified with the use of laser scanning densitometry and expressed as a percentage of the control. Values are mean ± SEM. The number of animals in each group was 6. *p < 0.05, **p < 0.01, ***p < 0.001

As shown in Figure 3(A), CWSS significantly lowered the expressions of p-JNK in the hypothalamus at 0 min after CWSS. However, the levels of p-JNK was elevated to the normal level in the hypothalamus 15 and 30 min after CWSS. Similarly, in the pituitary, both 0 and 15 min after CWSS, the phosphorylation level of JNK was significantly decreased, whereas it was reversal to normal level at 30 min (Figure 3(B)). CWSS also reduced the phosphorylation level of JNK in the adrenal gland at 0 min and maintained to 30 min (Figure 3(C)).

### Effect of CWSS on the expression of p-P38 in the HPA axis

**Figure 4.**
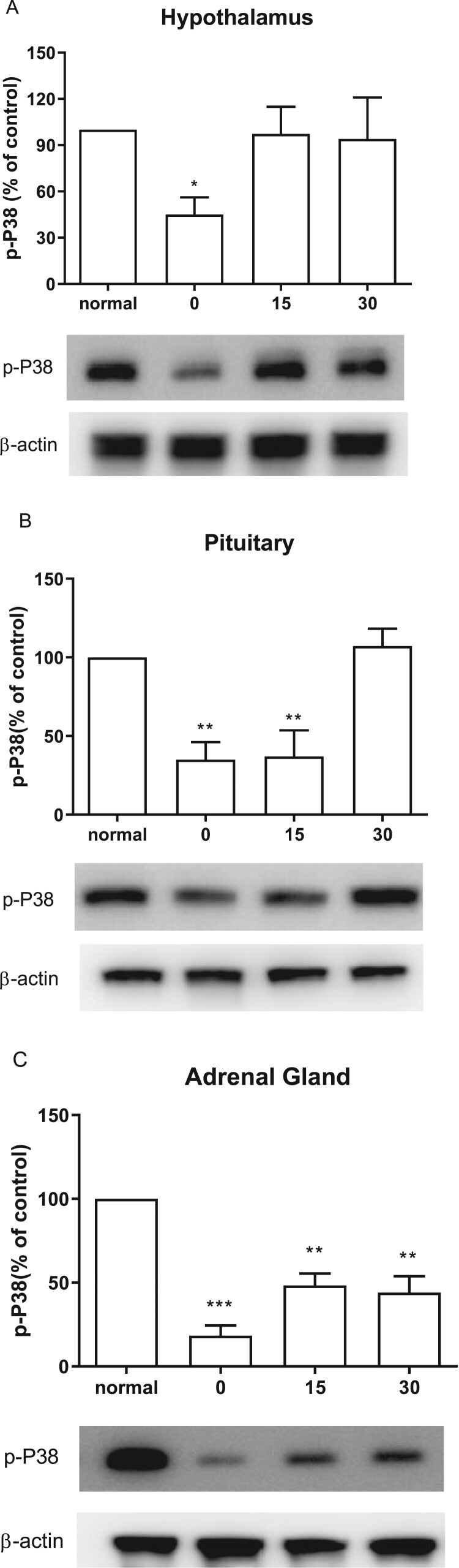
Effect of acute CWSS on P38 protein expression in the HPA axis. The protein p-P38 expression in the hypothalamus (A), in the pituitary (B), and the adrenal gland (C) were analyzed by Western blot. *β*-Actin (1:1000 dilution) was used as an internal loading control. Signals were quantified with the use of laser scanning densitometry and expressed as a percentage of the control. Values are mean ± SEM. The number of animals in each group was 6. *p < 0.05, **p < 0.01, ***p < 0.001

We further examined the changes of p-P38 after CWSS in the HPA axis. Expression level in the HPA axis of p-P38 protein was immediately decreased at 0 min after CWSS (Figure 4). As shown in Figure 4(A), the p-P38 expression level in the hypothalamus was reversal to normal level 15 min after CWSS. In Figure 4(B), the decreased p-P38 expression was maintained at 15 min and then back to normal level at 30 min after CWSS. However, in the adrenal gland, even after 30 min, the decreased expression level of p-P38 was still lower than that of the normal level (Figure 4(C)).

### Effect of CWSS on the expression of p-mTOR in the HPA axis

**Figure 5.**
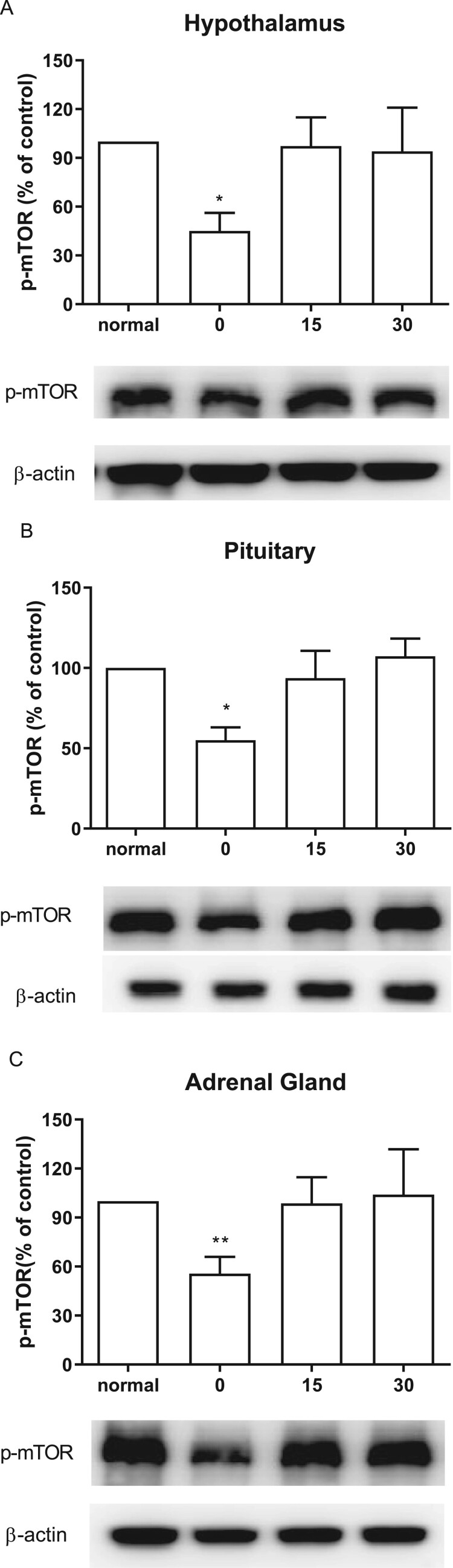
Effect of acute CWSS on mTOR protein expression in the HPA axis. The protein p-mTOR expression in the hypothalamus (A), in the pituitary (B), and the adrenal gland (C) were analyzed by Western blot. *β*-Actin (1:1000 dilution) was used as an internal loading control. Signals were quantified with the use of laser scanning densitometry and expressed as a percentage of the control. Values are mean ± SEM. The number of animals in each group was 6. *p < 0.05, **p < 0.01, ***p < 0.001.

We also inspected the changes of p-mTOR expression level after CWSS. As shown in Figure 5, in the HPA axis, including the hypothalamus, the pituitary, and the adrenal gland, the mTOR phosphorylation level was immediately lowered after CWSS. However, after 15 min up to 30 min, the suppressed level of p-mTOR induced by CWSS was reversed to the normal level.

### Effect of CWSS on the expression of p-AMPKa1 in the HPA axis

**Figure 6.**
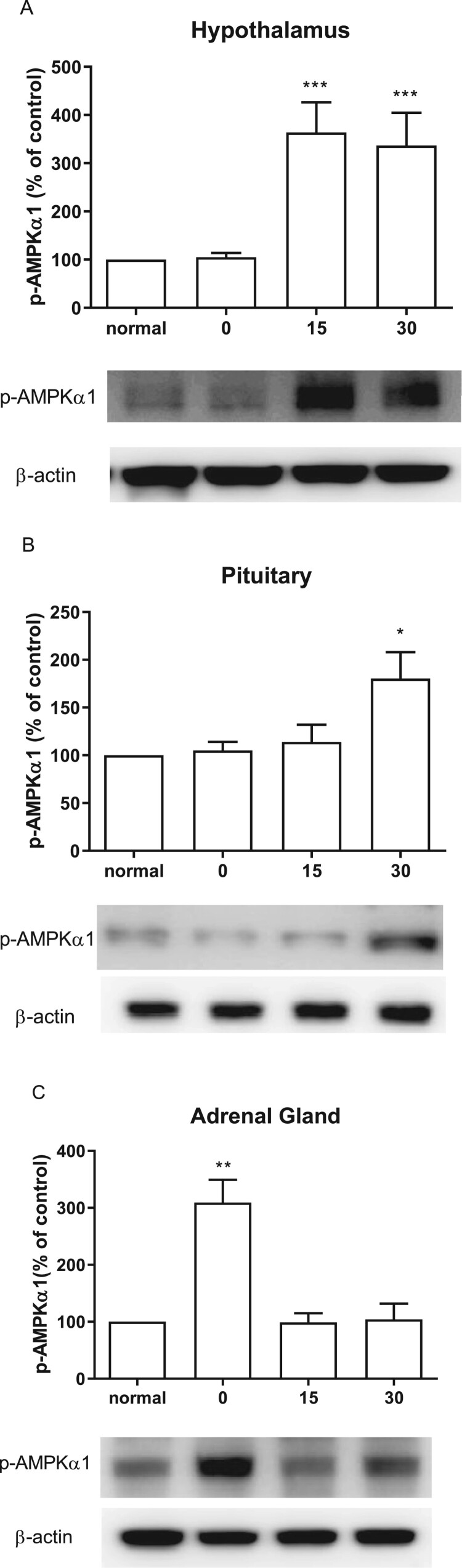
Effect of acute CWSS on AMPKα1 protein expression in the HPA axis. The protein p-AMPKα1 expression in the hypothalamus (A), in the pituitary (B), and the adrenal gland (C) were analyzed by Western blot. *β*-Actin (1:1000 dilution) was used as an internal loading control. Signals were quantified with the use of laser scanning densitometry and expressed as a percentage of the control. Values are mean ± SEM. The number of animals in each group was 6. *p < 0.05, **p < 0.01, ***p < 0.001.

CWSS caused the up-regulation of the expression level of p-AMPKα1 in the HPA axis. In the hypothalamus, 15 min after CWSS, the expression of p-AMPKα1 was significantly increased and maintained up to 30 min (Figure 6(A)). Similarly, the expression of p-AMPKα1 was mildly elevated at 30 min after CWSS in the pituitary (Figure 6(B)). However, in the adrenal gland, CWSS immediately raise the expression level of p-AMPKα1 at 0 min, which was back to normal level 15 and 30 min after CWSS (Figure 6(C)).

### Effect of CWSS on the expression of p-AMPKα2 in the HPA axis

**Figure 7.**
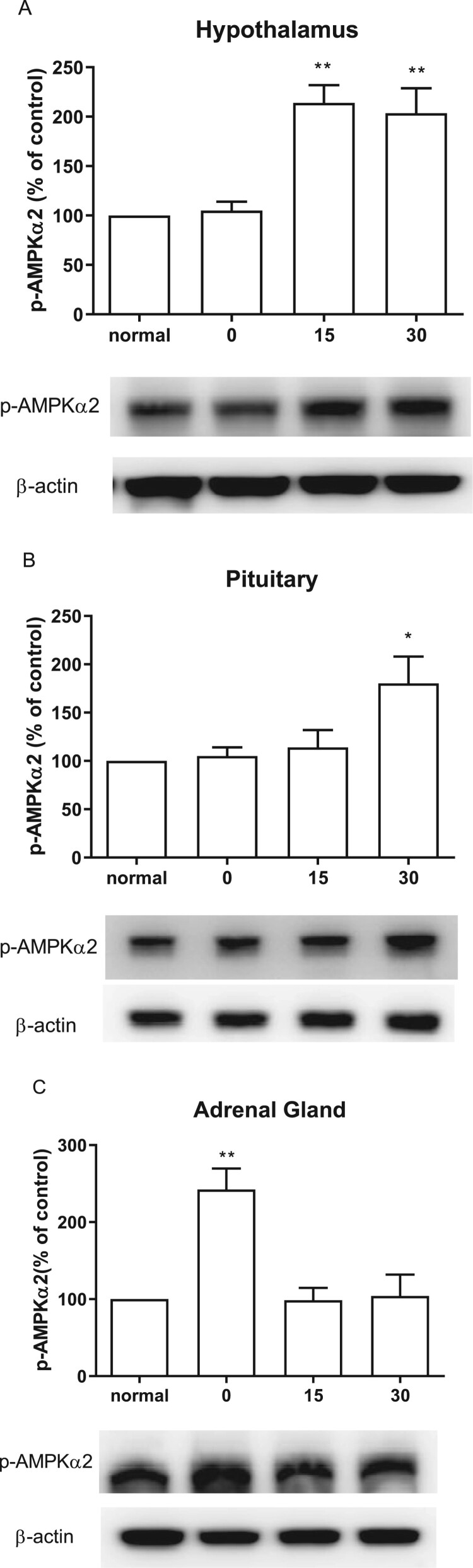
Effect of acute CWSS on AMPKα2 protein expression in the HPA axis. The protein p-AMPKα2 expression in the hypothalamus (A), in the pituitary (B), and the adrenal gland (C) were analyzed by Western blot. *β*-Actin (1:1000 dilution) was used as an internal loading control. Signals were quantified with the use of laser scanning densitometry and expressed as a percentage of the control. Values are mean ± SEM. The number of animals in each group was 6. *p < 0.05, **p < 0.01, ***p < 0.001.

Similarly, AMPKα1, AMPKα2 phosphorylation level was also elevated by the CWSS (Figure 7). As shown in Figure 7(A), the expression of p-AMPKα2 was remarkably increased at 15 min, which was maintained to 30 min after CWSS. In the pituitary, the expression of p-AMPKα2 was elevated at 30 min after CWSS (Figure 7(B)). Differently, CWSS raise the expression level of p-AMPKα2 quickly at 0 min, and then the increased level of p-AMPKα2 was reversed to normal level 15 and 30 min after CWSS in the adrenal gland (Figure 7(C)).

## Discussion

In previous studies, we and others have observed that plasma CORT is increased in different types of animal stress models (Gong et al. 2015; Suh et al. 2016). In the present study, we observed that CWSS increase corticosterone secretion at 0 min, and peaked at 15 min, with maintaining peak-level at 30 min. Gong et al. (2015) reported that, during forced swimming or heat stress, the level of CORT reaches maximum at 40 min of stress. Our previous study found that CWSS also causes the elevation of the blood glucose level (Suh et al. 2016). Thus, we suggested that after CWSS, the increase of CORT and blood glucose contribute to coping with their adaptation to stress. The main effect of these series responses of coordinate metabolism is the aim to maintain homeostasis after stress.

Corticotropin-releasing hormone (CRH) plays a key role in adjusting the basal and stress-activated HPA axis. MAPK signaling pathways, especially ERK, triggered by CRH through the CRH receptor 1 plays an essential role in CRH action in pituitary corticotrophs (Bonfiglio et al. 2011). On the contrast, in the current study, we determined that within 15 min after CWSS, the expression level of p-ERK is lower than the normal level. At the same time, the CORT level was gradually increasing. Zheng et al. (2008) demonstrated that after the cold exposure (−15°C, 2 h), the p-ERK level decreases immediately, and then increases drastically to peak level after 30 min in the rat brain, which partially support our results. Based on these pieces of evidence, we interfered that, during 0–15 min after CWSS, the expression level of p-ERK was regulated by stress stimuli directly. However, after the CORT level reached the maximum, the negative feedback became the main factor in the protein expression. Moreover, it is well known that a high concentration of GCs, such as CORT, exert negative feedback effects both on the activation of CRH neurons of the hypothalamus and on the secretion of adrenocorticotropic hormone (ACTH) in the pituitary (Vandenborne et al. 2005; Raubenheimer et al. 2006; Weiser et al. 2011). Although we did not characterize the exact mechanism, we hypothesized that the negative feedback may result in the different patterns of ERK phosphorylation levels among the adrenal gland, the hypothalamus, and the pituitary.

As another two members in the MAPKs family, JNK and p38 were reported to play a significant role in various brain regions as signaling mediator of different types of physical and psychological stressors (Meller et al. 2003; Zheng et al. 2008). The capability of JNK and P38 to phosphorylate GC receptor implied their regulatory role in the central nervous system (CNS) stress response. In the present study, we found that, in the hypothalamus, p-JNK and p-P38 expression levels are decreased transitorily and then returned to normal levels. However, some other studies demonstrated that the levels of p-JNK and p-p38 are significantly increased in the cold (4°C for 12 h) stress group (Xu et al. 2019) or in the forced swimming (25°C for 15 min) group (Shen et al. 2004). Accordingly, we speculated that these differential effects of stress might result from the differential origins of animal or the types of stress.

AMPK/mTOR pathway is essential to re-establish homeostasis after stress stimuli. The AMPK signaling pathway responds to increased AMP and ADP concentrations within the cell by dampening anabolic pathways and promoting catabolic pathways that replenish the ATP supply (Hotamisligil and Davis 2016). In support of this point, we demonstrated that the expression level of AMPK is increased after CWSS, whereas the level of mTOR was downregulated in CWSS mice. Some previous studies also determined that acute stress leads to phosphorylation of AMPK activated and phosphorylation of mTOR inhibited (Zhou et al. 2017; Jiang et al. 2019).

In conclusion, the present study clearly shows that CWSS actives the HPA axis and increase plasma CORT. Several signaling molecules, such as ERK, JNK, P38, AMPKα1, p-AMPKα2, and mTOR, were appeared to be involved in the activation of the HPA axis and CORT secretion induced by CWSS.
